# Identification of immune cell infiltration and diagnostic biomarkers in unstable atherosclerotic plaques by integrated bioinformatics analysis and machine learning

**DOI:** 10.3389/fimmu.2022.956078

**Published:** 2022-09-23

**Authors:** Jing Wang, Zijian Kang, Yandong Liu, Zifu Li, Yang Liu, Jianmin Liu

**Affiliations:** ^1^ Neurovascular Center, Changhai Hospital, Naval Medical University, Shanghai, China; ^2^ Department of Rheumatology and Immunology, Second Affiliated Hospital of Naval Medical University, Shanghai, China; ^3^ Department of Critical Care Medicine, Naval Medical Center of People's Liberation Army of China (PLA), Shanghai, China; ^4^ Department of Geriatrics, Navy 905th Hospital, Shanghai, China; ^5^ Department of Cardiovascular Surgery, Institute of Cardiac Surgery, Changhai Hospital, Naval Medical University, Shanghai, China

**Keywords:** carotid artery atherosclerosis, unstable atherosclerosis, immune infiltration, machine learning, biomarker

## Abstract

**Objective:**

The decreased stability of atherosclerotic plaques increases the risk of ischemic stroke. However, the specific characteristics of dysregulated immune cells and effective diagnostic biomarkers associated with stability in atherosclerotic plaques are poorly characterized. This research aims to investigate the role of immune cells and explore diagnostic biomarkers in the formation of unstable plaques for the sake of gaining new insights into the underlying molecular mechanisms and providing new perspectives for disease detection and therapy.

**Method:**

Using the CIBERSORT method, 22 types of immune cells between stable and unstable carotid atherosclerotic plaques from RNA-sequencing and microarray data in the public GEO database were quantitated. Differentially expressed genes (DEGs) were further calculated and were analyzed for enrichment of GO Biological Process and KEGG pathways. Important cell types and hub genes were screened using machine learning methods including least absolute shrinkage and selection operator (LASSO) regression and random forest. Single-cell RNA sequencing and clinical samples were further used to validate critical cell types and hub genes. Finally, the DGIdb database of gene–drug interaction data was utilized to find possible therapeutic medicines and show how pharmaceuticals, genes, and immune cells interacted.

**Results:**

A significant difference in immune cell infiltration was observed between unstable and stable plaques. The proportions of M0, M1, and M2 macrophages were significantly higher and that of CD8^+^ T cells and NK cells were significantly lower in unstable plaques than that in stable plaques. With respect to DEGs, antigen presentation genes (CD74, B2M, and HLA-DRA), inflammation-related genes (MMP9, CTSL, and IFI30), and fatty acid-binding proteins (CD36 and APOE) were elevated in unstable plaques, while the expression of smooth muscle contraction genes (TAGLN, ACAT2, MYH10, and MYH11) was decreased in unstable plaques. M1 macrophages had the highest instability score and contributed to atherosclerotic plaque instability. CD68, PAM, and IGFBP6 genes were identified as the effective diagnostic markers of unstable plaques, which were validated by validation datasets and clinical samples. In addition, insulin, nivolumab, indomethacin, and α-mangostin were predicted to be potential therapeutic agents for unstable plaques.

**Conclusion:**

M1 macrophages is an important cause of unstable plaque formation, and CD68, PAM, and IGFBP6 could be used as diagnostic markers to identify unstable plaques effectively.

## Introduction

Ischemic stroke is one of the major causes of death, functional limitations, and disabilities worldwide, accounting for more than 80% of 760,000 stroke cases that occur each year in the United States (1). Ischemic stroke is commonly caused by atherosclerosis, a chronic inflammatory disease with a progressive pathological process involving the accumulation of intimal plaque in the arterial wall (2). Despite the fact that atherosclerosis develops slowly and remains silent for decades, it can pose a life-threatening threat when plaque bursts or ruptures, leading to ischemic stroke and myocardial infarction (3). The stability of atherosclerotic plaques is a major factor in the development of symptomatic stroke and increases the risk of ischemic stroke and acute coronary syndromes (4). Unstable plaques are characterized by large necrotic cores, thin fibrous caps, and speckled calcifications accompanied with increased inflammation (5). As unstable plaques are more likely to rupture and cause local thrombosis or embolism, early identification of unstable plaques and prevention of rupture or erosion are of clinical importance.

Immune cell infiltration within the vessel wall is closely associated with the stability and progression of atherosclerosis (6). Monocytes and macrophages participate in the engulfment of oxidized low-density lipoprotein. They also play an important role in inflammatory responses by secreting pro-inflammatory mediators, matrix-degrading proteases (MMPs), eventually causing cell death through necrosis or apoptosis. The death of macrophages leads to the release of lipids and tissue factors, resulting in a pro-thrombotic necrotic core, which is one of the fundamentals of unstable plaques (7). A significant body of experimental evidence has demonstrated that T helper (Th) 1 cells play a pro-atherogenic role, while regulatory T (Treg) cells play an anti-atherogenic role. Treg cells, on the other hand, can become pro-atherogenic (8). By controlling immune responses through cell–cell contact, antigen presentation, and cytokine production, B cells play a part in both systemic and local immune responses in atherosclerotic arteries (9). However, the specific characteristics of dysregulated immune cells and effective diagnostic biomarkers associated with plaque stability within atherosclerosis lesions are poorly understood.

Many gene expression profiles have been applied to investigate the immune cell distribution and molecular diagnostic biomarkers by using high-throughput sequencing technologies such as microarray, RNA-sequencing (RNA-seq), and single-cell RNA-sequencing (scRNA-seq). As a powerful tool for discovering important cell types and diagnostic markers, machine learning has been widely employed in many studies to identify relevant biomarker features and in classifying and validating biomarkers (10, 11) In the present study, we first estimated the quantity of 22 immune cell using the CIBERSORT approach and calculated differentially expressed genes (DEGs) between stable and unstable atherosclerotic carotid artery tissues, then used machine learning algorithms to filter essential cell types and hub genes, and finally validated key cell types and hub genes in clinical samples. This study aims to explore the key roles of immune cells and key genes in the development and progression of unstable plaques for the sake of providing new perspectives for disease diagnosis and treatment and the study of immune molecular mechanisms.

## Methods

### Data collection

The gene expression profiles of carotid atherosclerotic plaques in the training set and the validation set were downloaded from GEO database (http://www.ncbi.nlm.nih.gov/geo) and EBI database (https://www.ebi.ac.uk/services). The training set was obtained from GSE120521 (12), GSE41571 (13), GSE163154 (14), GSE111782 (15), and GSE43292 (16), and the validation set was obtained from E-MTAB-2055. Single-cell data of carotid atherosclerotic plaques were obtained from GSE155514 (17), including two asymptomatic patients and one symptomatic patient. The included datasets and the baseline clinical characteristics of atherosclerosis patients are provided in **Supplementary Table S1**.

### Clinical sample collection and histological stability measurement

Carotid atherosclerotic plaques were obtained from patients who had undergone carotid endarterectomy. Ethical approval was granted by the local ethics committee of Shanghai Changhai Hospital (Ethics approval number: CHEC2020-164). All patients were fully informed of the research and signed the informed consent forms. Clinical data for included patients could be obtained in **Supplementary Table S2**. Histological stability was defined according to a modified, well-defined, well-validated American Heart Association (AHA) atherosclerotic scoring system (18, 19). This scoring system grades the severity of hemorrhage, thrombus, lipid core, fibrous tissue, chronic plaque inflammation, chronic cap inflammation, acute plaque inflammation, acute cap inflammation, foam cells, neovascularity, and cap rupture to measure the overall stability of the plaques. The details of score points are shown in **Supplementary Table S3**.

### Data pre-processing

GSE120521, GSE41571, GSE163154, GSE111782, and GSE43292 were downloaded using the R package GEOquery. The probe expression matrix was then converted into a gene expression matrix using the platform annotation file. The array data expression matrix was normalized by robust multichip average (RMA). The average value was obtained if multiple probes corresponded to one gene. Then, we merged the five datasets and used the “ComBat” function in sva package to remove batch effects among the datasets. To estimate the batch effect in the merged data, principal component analysis (PCA) was used to visualize the data.

### Immune cell infiltration analysis by CIBERSORT

Based on the normalized gene expression data from the disease and control samples, the web tool CIBERSORT (http://CIBERSORT.stanford.edu/) was used to calculate immune cell infiltration and explore the disease immune microenvironment. The 22 immune cell genes (LM22) were used as the reference set. The number of permutations set was 1,000. A p-value < 0.05 in the CIBERSORT results was retained. The result of immune cell infiltration was visualized by ggplots and pheatmap packages and further subjected to PCA using the R package factoextra and FactoMineR. The correlation between various immune cells was calculated by Spearman analysis, and the result of immune cell infiltration correlation was visualized by using corrplot package.

### Differentially expressed genes analysis

The DEG analysis on unstable vs. stable plaques was performed by using t model.matrix, lmFit, contrasts.fit, eBayes, and topTable in R package limma. The threshold for DEGs was |log fold change (FC)| > 1.5 and false discovery rate (FDR) < 0.05. The results were visualized in volcano plots and heatmaps using the R packages ggplot and pheatmap.

### Functional enrichment analysis of DEGs

GO functional enrichment and KEGG enrichment analysis were performed using the R package ClusterProfiler. The GO analysis consisted of three main components, including biological process, cellular component, and molecular function. To ensure the reliability of the enrichment results, we used Benjamini–Hochberg FDR to correct the p-value for multiple hypothesis testing, and FDR < 0.05 indicated that the enrichment was statistically significant.

### Least absolute shrinkage and selection operator regression and random forest analysis

A least absolute shrinkage and selection operator (LASSO) regression prediction model was constructed by using the “cv.glmnet” function in R package glmnet. The parameters used in the LASSO analysis were alpha = 1 and nlambda = 1,000, and lambda.min was chosen as the optimal lambda. The random forest analysis was performed using the “RandomForest” function. The minimum error was chosen as the mtry node value, and the value of the image that tended to be stable was chosen as the ntree. The top 10 key difference immune cells and top 30 key DEGs were selected based on the feature weights mean decrease accuracy (MDA) and mean decrease Gini (MDG), respectively. The union of differential immune cells and differential genes obtained by random forest analysis algorithm and LASSO regression prediction model was used to screen key differential immune cells and key DEGs.

### scRNA-seq data analysis

The Seurat package (20) was used to analyze single-cell data. Low-quality cells were defined as those with <200 unique molecular identifiers (UMIs) or mitochondrion-derived UMI counts of higher than 20%. Gene expression matrixes were normalized and scaled to acquire linear conversion for the remaining high-quality cells using the “NormalizeData” function and the “ScaleData” function. For PCA, the top 2,000 variable genes were selected, and the 30 most important principal components were employed for cluster analysis. The batch effects were subsequently eliminated by combining single-cell data from several samples using the Harmony package’s “Runharmony” function (21). The clusters were shown using uniform manifold approximation and projection (UMAP). Cell types were annotated based on the expression of known markers such as T cells (CD3D, CD4, and CD8), B cells (CD79A), SMC (MYH11, TNFRSF11B, and LUM), myeloid cells (FCN1, CD68, IL1B, and CD163), endothelial cells (VWF and DYSF), mast cells (TPSB2), and fibroblast (THY1). To assess the stability score and instability scores of different cell subclusters, we included low and high expressed genes of unstable plaques, calculated the scores using the “AddmoduleScore” function, and visualized with violin plots.

### Deconvolution of bulk RNA-seq data based on scRNA-seq reference data

To establish the proportions of our defined 16 major cell types in bulk RNA-seq data, BisqueRNA (v1.0.5) (22) and MuSic (v0.2.0) (23) were used to deconvolute the cellular composition. Briefly, the expression matrix and cell-type annotations were extracted in the scRNA-seq data to create a reference signature matrix. The corrected bulk RNA-seq matrix and reference signature matrix were performed by deconvolution analysis using “ReferenceBasedDecomposition” function in Bisque and “music_prop” in MuSic with default parameters. The estimated cell fractions were visualized in bar plots.

### The regulatory network of immune-related genes and immune cells

We calculated Spearman correlations based on key difference gene expression and key difference immune cells, selected interactions with absolute values of correlations > 0.1 to construct regulatory networks, and considered statistically significant pairs of key difference genes and key difference immune cells. The network was visualized using Cytoscape software (version 3.8.2). The intersection of the top 30 genes of the three algorithms was selected as the core gene.

### miRNA regulatory gene network construction

Seven bioinformatics tools were utilized to predict miRNA target genes, including miRanda (http://www.microrna.org/), RNA22 (https://cm.jefferson.edu/rna22/), TargetScan (http://www.targetscan.org/vert_72/), PITA(https://genie.weizmann.ac.il/pubs/mir07/mir07_data.html, PicTar (https://pictar.mdc-berlin.de/),DIANA-microT(http://diana.imis.athena-innovation.gr/DianaTools/index.php), and miRmap (https://mirmap.ezlab.org/). The miRNA target genes were selected when it had been predicted by at least five tools.

### Histology and immunohistochemistry

Fresh carotid atherosclerotic plaque samples were fixed in 4% neutral formaldehyde overnight and embedded in paraffin. Five-millimeter-thick serial sections were cut from the samples. In order to retrieve antigen from the slides, they were deparaffinized and microwave heated in citrate buffer (pH 6.0). Using 3% hydrogen peroxide, endogenous peroxidase activity was quenched after gradual cooling. The primary antibodies were incubated at 4°C overnight after protein blockage with 3% bovine serum albumin (BSA) for 30 min. Slides were incubated with secondary antibodies for 60 min at room temperature after being washed with phosphate-buffered saline (PBS). A diaminobenzidine-based color development followed by hematoxylin counterstaining was performed on the slides. The following primary antibodies were used: CD68 (Abcam, U.K.), PAM (Abcam, U.K.), and IFGBP6 (Abcam, U.K.).

### IHC score analysis

We used the Densito quant module in Quant Center 2.1 software to quantify the immunohistochemistry (IHC) score of the target region for each spot on each chip separately. IHC score =∑(PI×I)=(percentage of cells of weak intensity×1)+(percentage of cells of moderate intensity×2)+percentage of cells of strong intensity×3. PI indicates the percentage of positive signal pixel area; I represents the coloring intensity.

### Potential therapeutic drugs for unstable atherosclerotic plaques

Based on the core genes, The DGIdb database (https://www.dgidb.org/) of gene–drug interaction data was used to identify potential therapeutic drugs, and a Sankey diagram was used to depict the interactions between drugs, genes, and immune cells.

### Statistical analysis

Statistical analyses were mainly performed using R (version 4.0.4) and GraphPad Prism (version 8.0.1). Wilcoxon test was used to estimate the expression differences between two groups. Correlations between stability score and IHC scores were calculated using Spearman’s rank correlation.

## Results

### Immune cell infiltration in unstable and stable carotid artery plaques

Four microarray raw datasets and one RNA-seq dataset containing a total of 77 cases of unstable and 67 cases of stable carotid atherosclerosis were selected as the training dataset for the study of immune cell infiltration and DEG analysis. The basic information of the selected datasets is shown in **Supplementary Table S1**. Through gene expression profiling and PCA, we observed that there were baseline batch differences in the merged datasets (**Supplementary Figures S1A, B**). Then, we used “ComBat” algorithm to correct the batch effect so as to increase the analysis power in the following analysis. After applying the batch-correction methods, the batch effects were all eliminated to some extent (**Supplementary Figures S1C, D**).

**Figure 1:**
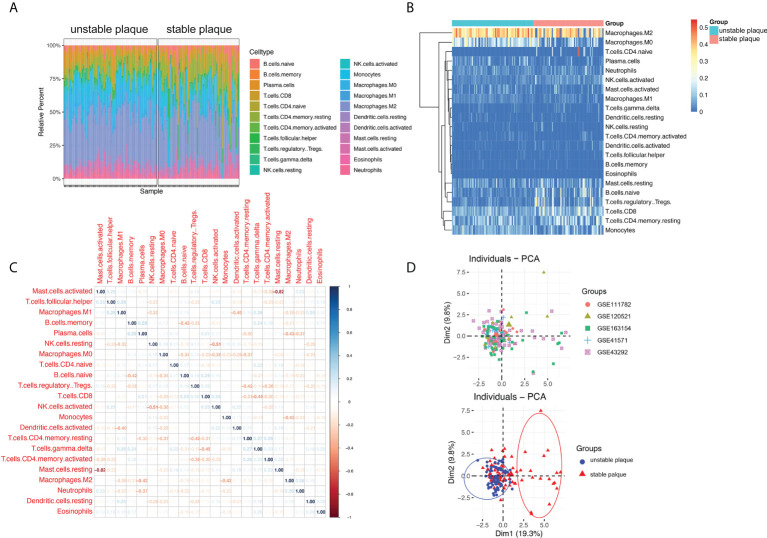
Immune cell infiltration in unstable and stable plaques. **(A)** Bar plot showing the composition of 22 types of immune cells across samples. **(B)** Heatmap of the composition of 22 types of immune cells across samples, colored by normalized relative abundance. **(C)** Correlation heatmap of 22 types immune cells in ruptured plaque samples. Blue indicates positive correlation, and red indicates negative correlation. **(D)** Principal component analysis plot of immune cells infiltration, colored by dataset (upper) and plaque stability (lower).

To study immune cell infiltration in unstable and table carotid artery plaques, the corrected expression matrix (including five datasets) was subjected to CIBERSORT to estimate the abundances of infiltrating immune cells in a mixed cell population (24). The results showed that M2 macrophages, CD8^+^ T cells, resting mast cells, naive B cells, Tfh cells, M0 macrophages, and M1 macrophages were the main immune cells that infiltrated the plaque (**Figures 1A, B**). The correlation between immune cells in unstable carotid plaques was further investigated. In mast cells, NK cells, and CD4^+^ memory T cells, the proportion of resting population was negatively correlated with that of the corresponding activated population (**Figure 1C**). The proportion of M1 macrophages was negatively correlated with that of NK and activated DC cells and positively correlated with that of gdT and resting DC cells. The proportion of M2 macrophages was negatively correlated with that of plasma and monocytes (**Figure 1C**). Based on the results of immune cell infiltration, unstable and stable plaques were clearly separated in the PCA plot, suggesting a significant difference in immune cell infiltration between unstable and stable plaques (**Figure 1D**).

**Figure 2:**
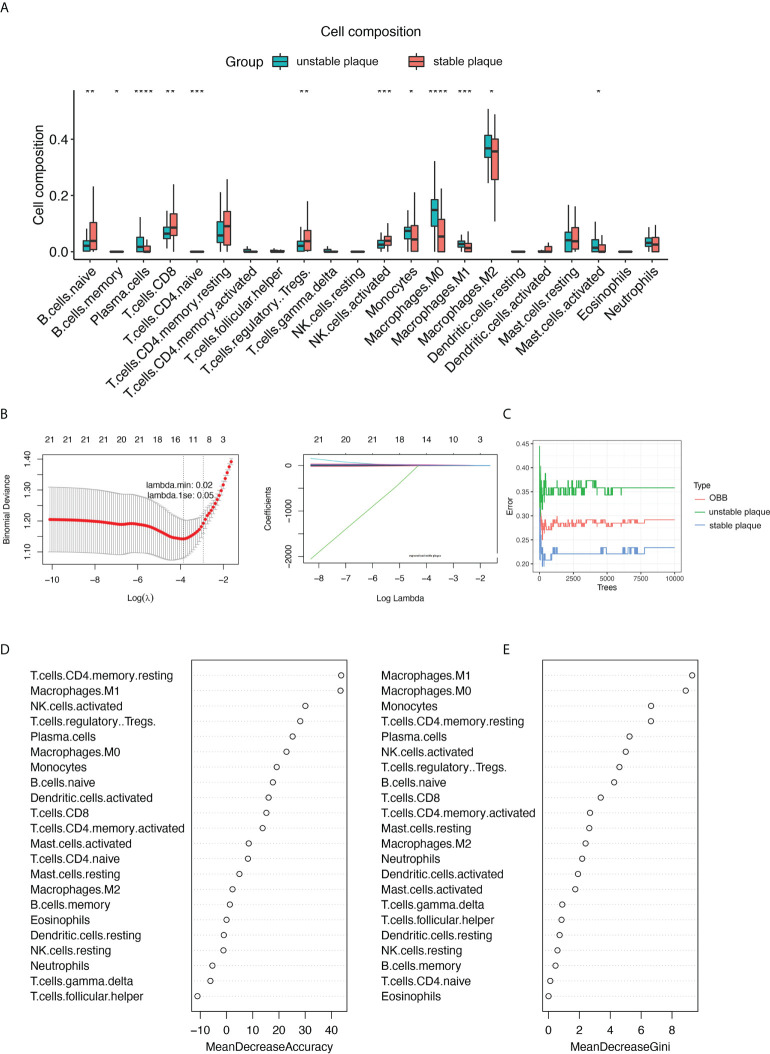
**|**Identification of key immune cell types associated with the stability of carotid plaques. **(A)** Identifying the significantly different infiltrates of immune cells in unstable and stable plaques by Wilcoxon test. **(B)** LASSO regression was conducted to analyze the different infiltrates of immune cells in unstable and stable plaques. **(C–E)** RandomForest was conducted to analyze the different infiltrates of immune cells in unstable and stable plaques. Key immune cell types associated with the stability of carotid plaques were identified by mean decrease accuracy **(D)** and mean decrease Gini **(E)**. *p < 0.05; **p< 0.01; ***p < 0.001; ****p < 0.0001.

Subsequently, we used the Wilcoxon test to identify differential immune cells between unstable and stable plaques in the merged dataset (**Figure 2A**) and individual datasets (**Supplementary Figure S2**). A total of 12 immune cell types were significantly different in unstable and sable plaques in the merged dataset (**Figure 2A**). For example, the proportion of M0, M1, and M2 macrophages in unstable plaques was significantly higher than that in stable plaques, and the proportion of CD8^+^ T cells and NK cells in unstable plaques was significantly lower than that in stable plaques (**Figure 2A**). LASSO regression and random forest algorithms were further applied to identify disease critical cell types. Using LASSO regression, we identified 12 immune cell types associated with plaque stability (**Figure 2B**). In the random forest algorithm, top 10 immune cells were selected as the key immune cell types based on the feature weights of MDA and MDG (**Figures 2C–E**). M1 macrophages and M1 macrophages, and resting CD4^+^ T memory cells were the top-ranked cell types by the two feature weights (**Figures 2D, E**). Based on the union of LASSO and random forest algorithms, we identified a total of 10 immune cell types associated with the plaque stability, including CD8^+^ T cells, naive B cells, monocytes, plasma cells, Tregs, activated NK cells, M0 macrophages, M1 macrophages, M2 macrophages, and activated mast cells.

### DEGs in unstable and table carotid artery plaques

**Figure 3:**
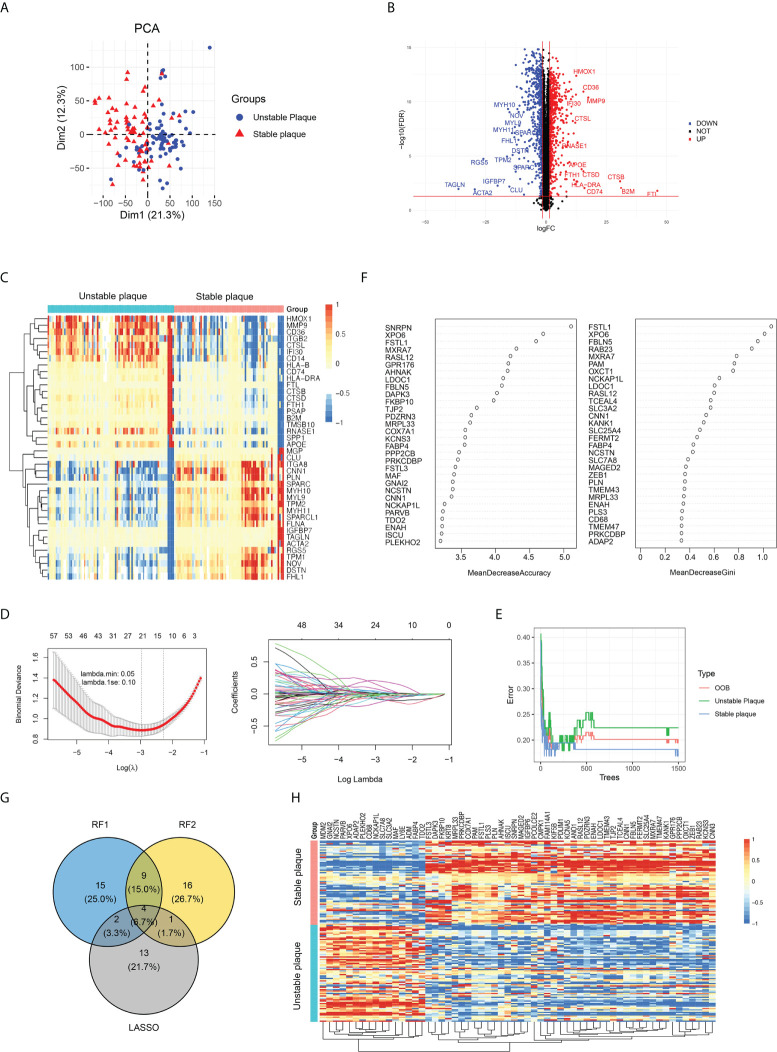
Identification of key differentially expressed genes (DEGs) between unstable and stable plaques. **(A)** PCA plot of gene expression profile, colored by unstable and stable plaque groups. **(B)** Volcano map of DEGs in unstable plagues compared to stable plagues. The significantly upregulated DEGs (FDR < 0.05 and a log2FC>1.5) were labeled in red, and the significantly downregulated DEGs (FDR < 0.05 and a log2FC<−1.5) were labeled in blue. **(C)** The expression of top 40 DEGs was visualized by heatmap. **(D)** LASSO regression was conducted to analyze the DEGs in unstable plague and stable plague groups. **(E, F)** RandomForest was conducted to analyze the DEGs in unstable plague and stable plague groups. **(G)** Venn diagram was conducted to obtain the intersection of the key DEGs screened by the two methods. **(H)** Expression of key DEGs was visualized by heatmap.

In addition to the immune cell infiltration analysis, we also explored the gene expression profiles between unstable and table carotid arterial plaques. The corrected expression matrix (including five datasets) was performed by PCA, and there was a significant group-bias clustering for the unstable and stable carotid arterial plaques, indicating that they had distinct gene expression profiles (**Figure 3A**). To identify unstable plaque-related genes, we performed DEG analysis and identified 1,139 DEGs, of which 600 genes were significantly upregulated and 539 genes were significantly downregulated in unstable plaques compared to stable plaques (**Figure 3B**; **Supplementary Table 4**). The expression of antigen presentation genes (CD74, B2M, and HLA-DRA), inflammation-related genes (MMP9, CTSL, and IFI30), and fatty acid-binding protein (CD36 and APOE) were elevated in unstable plaques, while the expression of smooth muscle contraction genes (TAGLN, ACAT2, MYH10, and MYH11) were decreased in unstable plaques (**Figures 3B, C**), suggesting that immune cell infiltration, lipid deposition, and decreased smooth muscle contractility were important factors contributing to plaque instability.

GO enrichment analysis revealed that DEGs were mainly enriched in “neutrophil activation involved in immune response,” “extracellular matrix organization,” “cell–substrate junction,” and “collagen binding” (**Supplementary Figure S3A**). DEGs in KKGG pathway were mainly enriched in the “focal adhesion” and “extracellular matrix (ECM)–receptor interaction pathways” (**Supplementary Figure S3B**). These findings suggested that plaque instability was primarily caused by immunological response, extracellular matrix, and cellular adhesion molecules. We further utilized LASSO regression and random forest algorithms to screen key DEGs. Using LASSO regression analysis, we screened 20 key DEGs (**Figure 3D**). Using the random forest algorithm, we selected the top 30 genes as the key DEGs based on the feature weights MDA and MDG (**Figures 3E, F**). Combining the above methods, we screened a total of 60 key genes associated with plaque instability, including 16 upregulated genes and 44 downregulated genes in unstable plaques vs. stable plaques (**Figures 3G, H**).

### Contribution of cell subsets to atherosclerotic plaque instability

**Figure 4:**
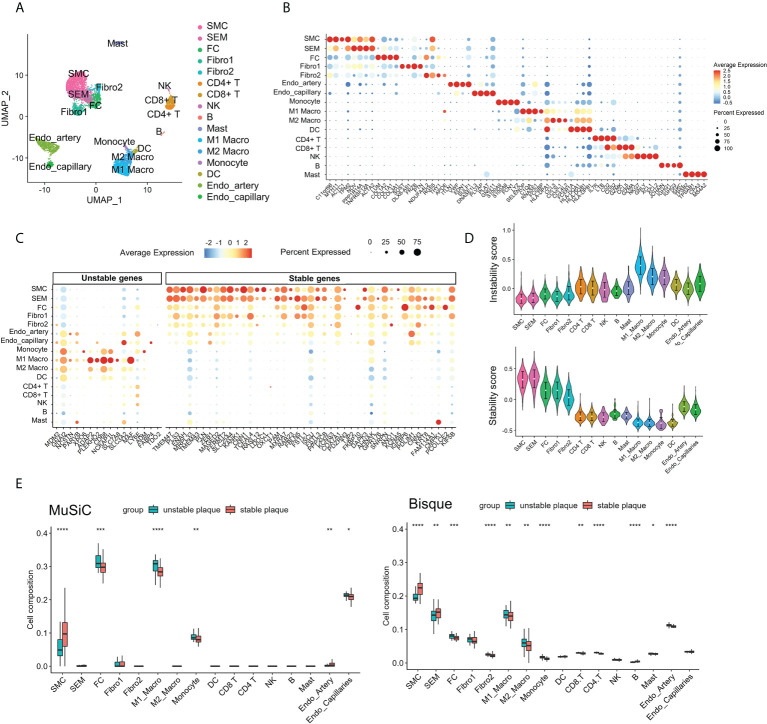
Contribution of M1 macrophages to atherosclerotic plaque instability. **(A)** UMAP projection showing the single-cell atlas of carotid atherosclerotic plaques. **(B)** Dot plot displaying the fractions of expressing cells (dot size) and mean expression level in expressing cells (dot color) of marker genes (rows) across clusters. **(C)** Dot plot showing the expression of DEGs in different clusters of carotid atherosclerotic plaques. **(D)** Violin plots showing the instability score (upper) and stability score (lower) in atherosclerotic plaque clusters. **(E)** Immune cell infiltration in unstable and stable plaques estimated by Music and BisqueRNA. *p < 0.05; **p< 0.01; ***p < 0.001; ****p < 0.0001.

To identify cell subsets that expressed genes associated with plaque instability, we collected scRNA-seq data of carotid plaques. Following quality control, we obtained 7,186 high-quality single-cell data, upon which we performed normalization, unsupervised dimensionality reduction, and graph-based clustering (**Figure 4A**). Annotations of cell types were based on canonical markers, such as ACAT2 for smooth muscle cell (SMC), CD3D for T cells, CD79A for B cells, and CD68 for macrophages (**Supplementary Figure S4**). We identified a total of 16 cell subsets including SMC subsets, endothelial cell subsets, myeloid cell subsets, and lymphocyte subsets. We also identified an intermediate state of SMC, termed “SEM” cells and fibrochondrocytes (FC) in the plaques based on the expression of TNFSF11B and LUM (**Supplementary Figure S4**). The marker genes for each subpopulation were calculated, and the results are shown in **Figure 4B**. We next explored the expression levels the DEGs across cell subsets. It was found that the stable plaque-associated genes were mainly expressed in SMC and SEM cells such as PLN, CNN1, and SLC25A4. In contrast, unstable plaque-associated genes were mainly expressed in macrophages including ADAP2 and MAF. We further constructed instability scores and stability scores based on the DEGs and found that myeloid cell subsets had high instability scores, with M1 macrophages having the highest score (**Figure 4C**). SMC, SEM, FC, and fibroblasts had high stability score, with SMC and SEM having the highest score (**Figure 4D**).

To further validate the immune infiltration of unstable plaques, we applied MuSiC and Bisque to deconvolute the cellular composition based on scRNA-seq marker genes. The results illustrated that the myeloid cells, especial M1macrophages, and FC cells were also elevated in unstable plaques. While SMC and SEM were downregulated in unstable plaques (**Figure 4E**). These findings indicated that loss of SMC and infiltration of macrophage subsets, especially M1 macrophages, were the most important risk factors contributing to plaque instability.

### Immune cells and DEGs synergistically affect plaque instability

**Figure 5:**
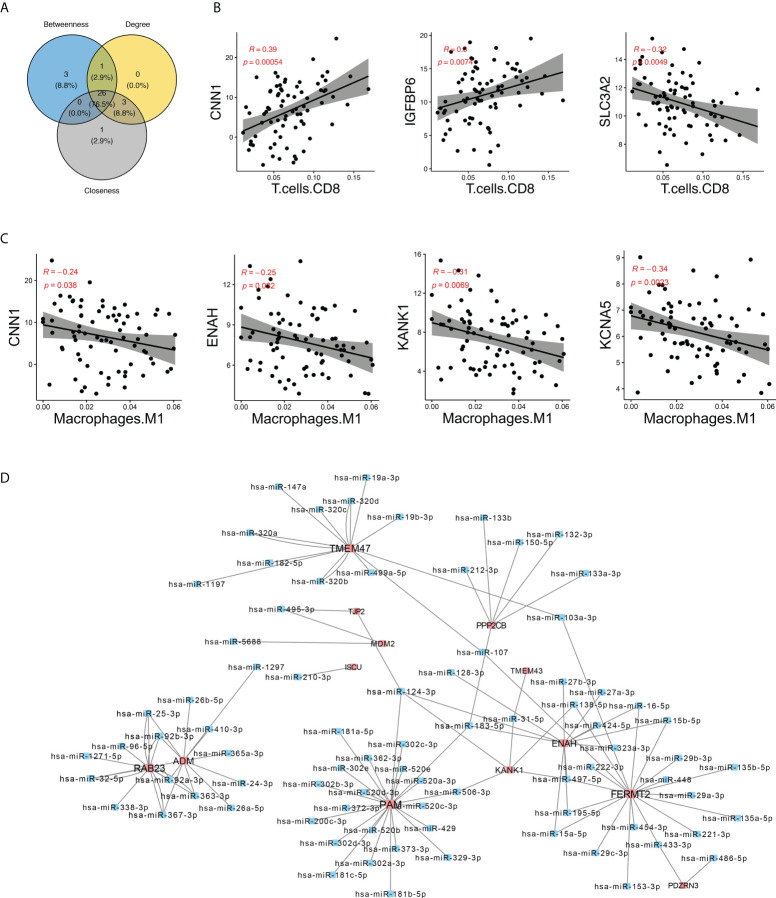
Identification of hub genes in the network. **(A)** Venn plot showing the intersection of genes identified by three algorithms. **(B, C)** Spearman’s correlation between hub DEGs CNN1, IGFBP6, and SLC3A2 and CD8^+^ T cells and M1 macrophages. **(D)** miRNA–genes network. The interactions between miRNA and target genes were predicted by seven bioinformatics tools; interactions appeared in at least five tools were visualized here. Genes are colored in red, while miRNA are colored in blue.

To determine the interaction relationship between DEGs and differential immune cells, we further calculated their Spearman correlations (**Supplementary Figure S5A**). Key DEGs with an absolute value of correlation >0.1 with immune cells were selected to construct the network (**Supplementary Figure S5B**). In this network, we selected the top 30 genes as core genes by three algorithms of degree, betweenness, and closeness; took the intersection of these three algorithms as the final core genes; and identified a total of 26 core genes (**Figure 5A**). We found that some core genes were significantly positively correlated with immune cells; for instance, IGFBP6, CNN1, and CD8^+^ T cells were significantly positively correlated, while SLC3A2 and CD8^+^ T cells were significantly negatively correlated (**Figure 5B**). CNN1, ENAH, KANG1, and KANG5 were significantly negatively correlated with M1 macrophages (**Figure 5C**). All these results suggested that immune cells may cooperate with differential genes to affect the plaque stability.

### Construction of miRNA regulatory network of core genes

Based on the data of gene interaction with miRNA from PITA, RNA22, miRmap, microT, miRanda, PicTar, and TargetScan databases, we further constructed an miRNA regulatory network of core genes and found that FERMT2, PAM, and TMEM47 were regulated by multiple miRNAs. For example, PAM was regulated by miR-181a, miR-181b, and miR200, and TMEM47 was regulated by miR495, miR320, and miR417 (**Figure 5D**). Knowing that miRNA plays a role in gene expression regulation and disease progression, the study of miRNA gene regulation network may help gain new insights to diseases diagnosis and treatment.

### CD68, PAM, and IGFBP6 genes are effective diagnostic markers of disease

**Figure 6:**
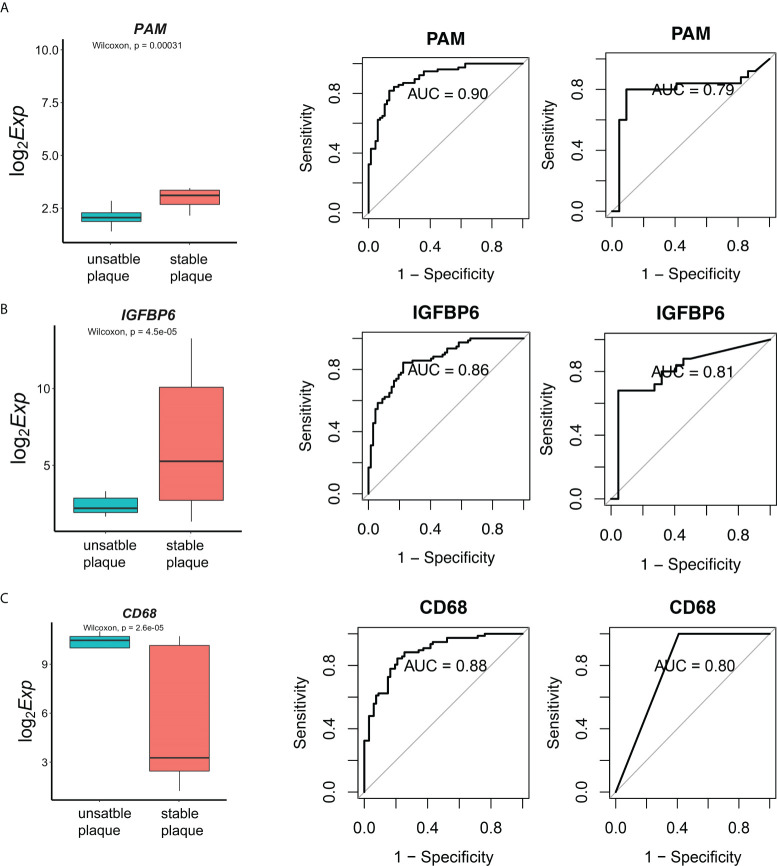
Diagnostic effectiveness of the biomarkers. **(A)** The E-MTAB-2055 dataset was used to validate the differential expression for gene PAM. Diagnostic effectiveness of the PAM by ROC analysis in the training set and validation dataset. **(B, C)** Expression levels and diagnostic effectiveness of IGFBP6 and CD68 in the training set and validation dataset.

To validate our above results, we further included another independent dataset E-MTAB-2055 with 24 unstable plaques and 23 unstable plaques as the validation dataset. A total of 28 genes were also differentially expressed in the validation dataset. In order to evaluate the diagnostic value of these genes, the receiver operating characteristic (ROC) curves were established, and the area under the ROC curve (AUC) was assessed. Fourteen genes were identified with high diagnostic efficacy (AUC>0.75) in both the training and validation sets (**Supplementary Figure S6**). Among the core genes that we identified, CD68 were the marker of myeloid cell subsets. There was a significant correlation between IGFBP6 and immune cells. PAM was regulated by more miRNAs. PAM was significantly downregulated in unstable plaque tissues in dataset E-MTAB-2055 (**Figure 6A**). The AUC value in training dataset and validation dataset was 0.90 and 0.79, respectively (**Figure 6A**), indicating that PAM had a good diagnostic performance for unstable plague. IGFBP6 was also significantly downregulated in unstable plaque tissues in the validation dataset (**Figure 6B**). IGFBP6 was also a good predictor for unstable plagues in both the training and validation datasets with an AUC value of 0.86 and 0.81, respectively (**Figure 6B**). In addition, CD68, the universal macrophage marker, was highly expressed in unstable plaques. The AUC value for CD68 in the training and validation dataset was 0.88 and 0.80, respectively (**Figure 6C**). These molecules were all good indicators of plaque stability.

**Figure 7:**
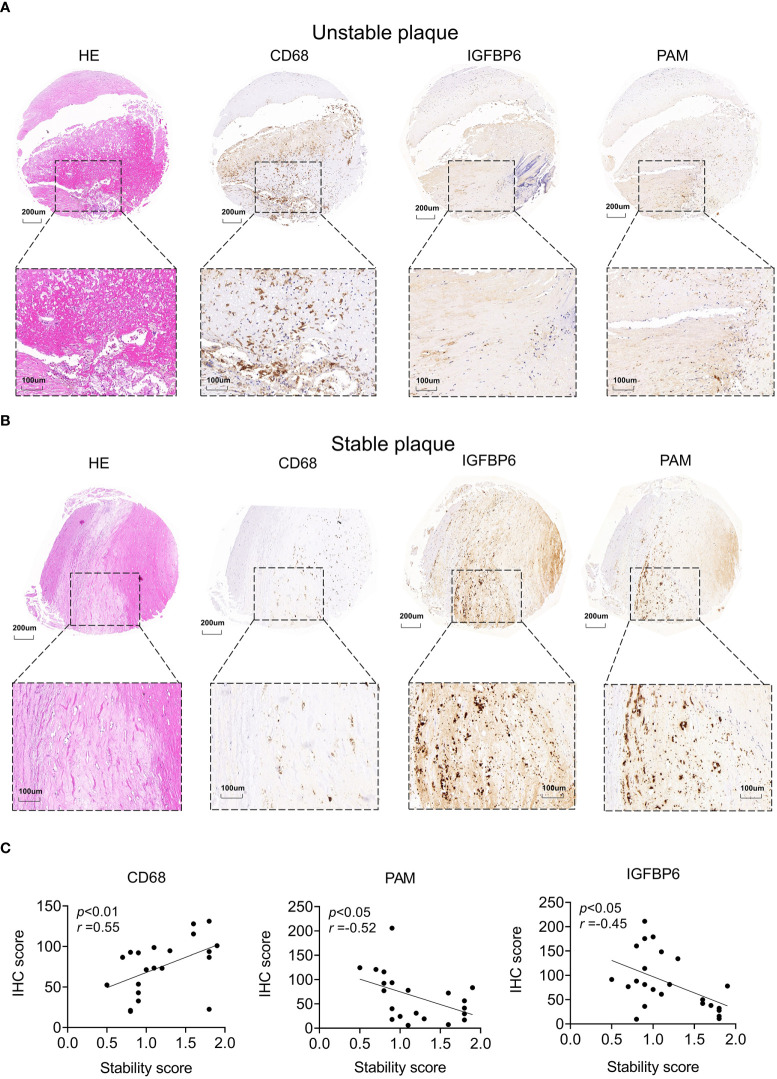
Potential biomarkers validated by clinical samples. **(A, B)** Representative images of hematoxylin–eosin and IHC staining for unstable **(A)** and stable **(B)** plaques. **(C)** Correlation analysis between stability and IHC score in atherosclerotic plaques. The significance was calculated by Spearman analysis.

To further validate these biomarkers for diagnosis of unstable plaques, we collected atherosclerotic samples and scored them based on AHA atherosclerotic scoring system (19) (**Supplementary Table 3**). Consistent with sequencing data, CD68 was highly expressed in unstable plaques and was downregulated in stable plaques (**Figures 7A, B**). In contrast, PAM and IGFBP6 were reduced in unstable plaques and were increased in stable plaques (**Figures 7A, B**). CD68 was significantly positively correlated with stability score, and PAM and IGFBP6 were negatively correlated with stability score (**Figure 7C**). Thus, these three genes could be reliable diagnostic predictors for unstable plaques.

### Potential therapeutic agents for unstable plaque treatment

**Figure 8:**
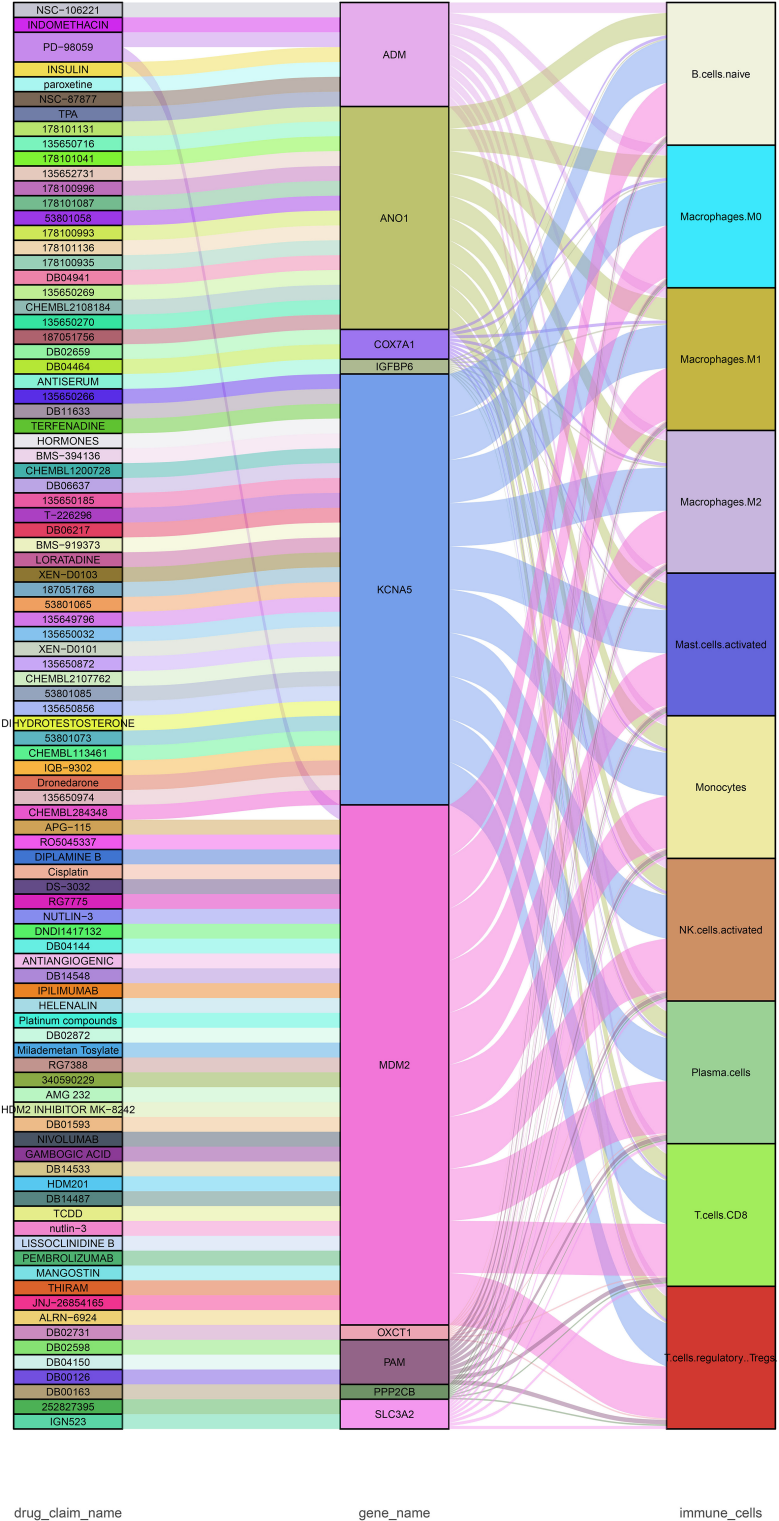
Sankey diagram showing the flow among drugs, genes, and immune cells. The drug–gene interaction was obtained from DGIdb (https://www.dgidb.org).

Based on key DEGs, we used the DGIdb database (https://www.dgidb.org/) of gene–drug interaction data to identify potential therapeutic drugs and demonstrate the interactions between drugs, genes, and immune cells (**Figure 8**; **Supplementary Table 5**). The 96 potential drugs were identified to target 10 genes. MDM2, KCNA5, and ANO1 had relatively abundant targeted drugs and were correlated with multiple immune cells, serving as the potential therapeutic targets for unstable plaques. Insulin, nivolumab, indomethacin, and α-mangostin were predicted to be potential therapeutic agents for unstable plaques, some of which have been proven to have clinical benefits for atherosclerosis or ischemic stroke.

## Discussion

The rupture of unstable plaque and the subsequent thrombus formation is one of the main causes of ischemic strokes. Prevention of cerebrovascular and cardiovascular adverse events requires identification of the biomarkers of patients at risk of stroke and viable therapeutic targets for unstable atherosclerosis plaque. In this study, we estimated the infiltrating immune cells and explored the potential biomarkers in unstable plaques in atherosclerosis patients based on bioinformatics analysis and machine learning, finding that M1 macrophages were an important cause of unstable plaque formation, and CD68, PAM, and IGFBP6 were also diagnostic markers for the early identification of unstable plaques.

Atherosclerosis is characterized by vascular inflammation, which adds to plaque instability (25). Despite decades of research, the immunological mechanisms underlying plaque instability remain largely unknown. Here, we obtained bulk RNA-seq data and microarray data of carotid atherosclerotic plaques, applied deconvolution methods, and compared the differences of immune cells between stable and unstable plaque samples. The results showed that M2 macrophages, M0 macrophages, M1 macrophages, and CD8^+^ T cells were the main immune cells that infiltrated the plaques, which was consistent with the finding of previous studies that T cells and macrophages represented the largest population of leukocytes in atherosclerotic plaques (26). The proportion of M0, M1, and M2 macrophages in unstable plaques were much higher than that in stable plaques, but the proportion of CD8^+^ T cells and NK cells was significantly lower than that in stable plaques, suggesting that macrophage infiltration is the main cause of unstable plaque formation. Monocyte-derived cells are recruited into the subendothelial region, where they evolve into mononuclear phagocytes, which consume the accumulated normal and modified lipoproteins, converting them into cholesterol-laden “foam cells.” Foam cells, a kind of macrophages, persist in plaques and contribute to disease progression. While lipoprotein clearance by macrophages is likely to be advantageous at the onset of this immune response, there is no negative feedback of absorption, resulting in these cells being engorged with lipids. The lipid metabolic imbalance may result in change in the macrophage phenotype and jeopardization of important immune activities (7).

Technological advances in characterizing molecular heterogeneity at the single-cell level enable us to better understand the biological diversity of cells present in atherosclerotic plaques (27). Alma Zernecke et al. (28) comprehensively provided authoritative information of the immune cell phenotypes in mouse model using scRNA-seq and mass cytometry. They identified 17 immune subsets including five macrophage subsets, namely, resident, inflammatory, Trem2+ foamy, interferon-inducible, and resembling cavity macrophages. We also investigated the immune cell phenotypes in patients with carotid atherosclerosis. In human atherosclerotic plaques, we did not identified neutrophils and ILC2 cells as those in mouse atherosclerotic plaques, probably because of species differences. We found that macrophages are the most abundant immune cell types and had distinct phenotypes. M1 macrophages, which were also detected in the mouse model, highly expressed inflammatory cytokines and had the highest instability score in atherosclerotic human aortas. This indicated that M1 macrophage cells could be the most important immune cells responsible for plaque formation and plaque instability. Inflammatory macrophages and resident macrophages also contributed to the plaque instability. Proinflammatory factors, such as tumor necrosis factor (TNF), interleukin (IL)-1, IL-12, and IL-23, and chemokines CXCL9, CXCL10, and CXCL11, might be secreted by M1 macrophages. High quantities of reactive oxygen species and nitric oxide are produced by these proinflammatory macrophages, contributing to the development of the inflammatory response. Cells expressing proinflammatory factors were preferentially distributed in shoulder regions that are more susceptible to rupture (29). It is worth noting that transcriptome may not comprehensively reflect the phenotype of myeloid cells. Future work could combine the power of cell surface phenotype (by CITE-Seq or CyTOF) and single-cell transcriptome analysis to provide a more comprehensive view of the immune cell infiltrate in atherosclerotic lesions.

We also explored the expression profiles between unstable and stable plaques. Further analysis indicated that the function of these upregulated genes in unstable plaques were associated with immune cells such as CD74, HLA-DRA, CSTB, and MMP9. While the downregulated genes in unstable plaques were correlated with SMC contraction genes such as TGALN, ACTA2, MYH11, and MYH10. GO and KEGG analysis also revealed that immunological response, extracellular matrix, and cellular adhesion molecules may be associated with plaque instability, suggesting that in addition to immune cells, SMCs and the extracellular matrix also affected plaque instability. SMCs secrete and deposit ECM proteins and are therefore thought to prevent the destabilization of atherosclerosis plaques. However, if SMCs undergo phenotypic transformation, they may release numerous MMPs that are capable of digesting ECM proteins (30). Our results also showed that SMCs had the highest stability score; the score was downregulated when SMCs turn into FC cells, indicating that SMC phenotypic transformation also affects plaque stability. Recently, lineage tracing and single-cell RNA sequencing profiling have revealed additional SMC plasticity in atherosclerosis, demonstrating that SMC may generate cells that look like foam cells, macrophages, mesenchymal stem cells, and osteochondrogenic cells (31–33). The functional influence of these phenotypes on plaque formation and stability and their relevance *in vivo* are worth further exploration.

Recent research has found that some inflammatory cytokines could be biomarkers for plaque vulnerability and major adverse vascular events, such as MCP-1 protein (34). CCL2, which encodes MCP-1, is also elevated in unstable plaques at the transcription level in our study. We further screened some potential effective diagnostic biomarkers of unstable plaques by LASSO regression and the randomized forest algorithm. These genes were further validated in the validation dataset and 14 unstable plaque-related genes (CD68, CNN3, FAM114A1, PKBP10, FSTL3, IGFBP6, KRT8, LY6E, OCCT1, PAM, PRKCDBP, RASL12, TMEM43, and TMEM47) were identified as good indicators for the diagnosis of unstable plaques. Some of these genes were reported to participate in the pathogenesis of atherosclerosis. For example, CNN3 is an actin filament-associated regulatory protein expressed in SMCs and multiple types of non-muscle cells. It can help inhibit actin-activated myosin ATPase and stabilize the actin cytoskeleton (35). FAM114A1 was recently identified as a candidate gene involved in coronary artery disease in a transcriptome−wide association study (36). FSTL3 was elevated in unstable plaques and could induce lipid accumulation and inflammatory response in macrophages through regulating CD36 and LOX-1 expression (37). Among the core genes that we identified, CD68 were the marker of myeloid cell subsets. IGFBP6 was significantly correlated with multiple immune cells; PAM was regulated by more miRNAs. These three genes were further validated with clinical samples to evaluate their expression levels and their diagnostic value.

In our study, machine learning has been used to screen critical genes and immune cells. Machine learning could provide better predictive performance than traditional statistical models, capturing complex interactions between predictors and nonlinear relationships between predictors and outcomes. Recent advancement in spatial transcriptomics technology has enabled us to explore the co-localization of different transcripts signals within the tissues (38). Several machine learning algorithms have been proposed to integrate spatial transcriptomics data and other data (39). Dongqing Sun et al. (40) presented STRIDE to decompose cell types from spatial mixtures by leveraging topic profiles trained from single-cell transcriptomics based on the machine learning method. Not only do this algorithm map rare cell types to spatial locations, but it also improves gene and domain localization. Hu et al. (41) developed the SpaGCN algorithm, which identifies genes with spatial patterns by integrating gene expression data, spatial location information, and histology images. Thus, machine learning technology could also be applied in spatial transcriptomic and is a highly promising perspective for atherosclerosis research. Machine learning has also been emerging as a highly effective method for outcome prediction, risk stratification biomarker discovery, and personalized medicine strategies in clinical research (42). For example, Terrada et al. (43) developed highly accurate diagnostic methods for the detection of atherosclerosis at a large scale. Ambale-Venkatesh et al. (44) applied machine learning methods to deep phenotyped datasets, giving accurate outcome predictions in cardiovascular event. Hand et al. (45) identified important features related to quantitative atherosclerosis characterization and patients at risk of rapid coronary plaque progression. In future research and medical practice, machine learning could help patient-centered precision medicine discovery and the development of the continuum from target validation to optimization of pharmacotherapy by exploiting information contained in diverse sources of big datasets such as “omics” data and by integrating advanced analytics into the practice of translational medicine.

We also predicted the potential therapeutic candidates for the unstable plaque. Some drugs are interesting. Insulin was predicted to target adrenomedullin (ADM) and treat unstable plaque in our study. It has previously been observed that insulin could improve cardiovascular outcomes by reducing plasma glucose and improving lipid profiles. Insulin also has pleiotropic effects, including anti-inflammatory, antithrombotic, and antioxidant characteristics. Insulin appears to be able to counteract the pro-oxidant effects of ambient hyperglycemia and glycemic fluctuation by inhibiting the activation of oxidative stress. However, insulin activities are still a point of contention when it comes to the risk of unfavorable cardiovascular events (46). The indomethacin was found to produce a 61% reduction in platelet thromboxane, indicating that it had a partial inhibitory effect on COX-1 *in vivo (* 47). In a retrospective study, nivolumab treatment was found to reduce atherosclerotic plaques. Strong PDL1 expression on dendritic cells within complicated plaques may govern previously unknown beneficial mechanisms (48). Physiological levels of dihydrotestosterone were reported to attenuate the progression of atherosclerosis in rabbits by suppressing intimal foam cell formation of macrophage partly *via* the suppression of LOX-1 expression (49). A meta-analysis reported that the risk of stroke or transient ischemic attack (TIA) reduced in patients with paroxysmal or persistent atrial fibrillation who received the antiarrhythmic agents dronedarone (50). α-Mangostin could decrease cholesterol and triglycerides and suppress the progression of atherosclerosis in Apoe−/− mice, possibly through M2 macrophage polarization (51). But as these drugs and targets are only predictions at the theoretical level, more animal experiments and clinical trials are needed to provide more evidence.

In conclusion, we identified M1 macrophage as an important cause of unstable plaque formation and CD68, PAM, and IGFBP6 as diagnostic markers for early identification of unstable plaques. These findings may provide a new strong scientific basis for the diagnosis and treatment of unstable atherosclerotic plaques.

## Data availability statement

The original contributions presented in the study are included in the article/**Supplementary Material**. Further inquiries can be directed to the corresponding authors.

## Ethics statement

The studies involving human participants were reviewed and approved by Shanghai Changhai Hospital ethics committee. The patients/participants provided their written informed consent to participate in this study. Written informed consent was obtained from the individual(s) for the publication of any potentially identifiable images or data included in this article.

## Author contributions

JL, YL, and ZL designed the overall research strategy. JW and YDL collected the clinical information and atherosclerotic plaque samples. JW and YDL performed the experiments. ZK performed the bioinformatics analysis. JW and ZK wrote the manuscript. JL, YL, and ZL participated in data discussion. All authors contributed to the article and approved the submitted version.

## Funding

This study was supported by the National Natural Science Foundation of China (Grant 82071278) and Clinical project of Shanghai Municipal Health Commission (Grant 20224Z0008).

## Acknowledgments

We thank Dr. Jianming Zeng (University of Macau) and all the members of his bioinformatics team, biotrainee, for generously sharing their experience and codes.

## Conflict of interest

The authors declare that the research was conducted in the absence of any commercial or financial relationships that could be construed as a potential conflict of interest.

## Publisher’s note

All claims expressed in this article are solely those of the authors and do not necessarily represent those of their affiliated organizations, or those of the publisher, the editors and the reviewers. Any product that may be evaluated in this article, or claim that may be made by its manufacturer, is not guaranteed or endorsed by the publisher.

## Supplementary material

Supplementary Figure 1 Data processing. **(A, B)** Box plot and principal component analysis (PCA) of expression profile across selected dataset before batch effect correction. **(C, D)** Box plot and principal component analyses of expression profile after batch effect correction.

Supplementary Figure 2 Immune infiltration in individual dataset. Identifying the significantly different infiltrates of immune cells in unstable and stable plaques by wilcoxon test

Supplementary Figure 3 GO and KEGG pathway enrichment analysis of the DEGs between unstable and stable plaques. **(A)** Dot plot of enriched GO termed for DEGs. **(B)** Dot plot of enriched KEGG pathway for DEGs.

Supplementary Figure 4 Cell Annotation by signature markers in scRNA-seq data. Heatmap of the expression levels of representative marker genes of the atherosclerotic plaque clusters. The color represents the gene expression levels.

Supplementary Figure 5 Correlation of DEGs and key immune cell types. **(A)** Heatmap shows Spearman correlation between DEGs and immune cells. **(B)** Network between key DEGs and key immune cell types. Red lines represent positive correlation while blue ones represent negative correlation. Immune cell types are colored in orange while genes are colored in blue.

Supplementary Figure 6 Potential biomarkers for unstable plaques. Diagnostic effectiveness of the potential biomarkers by ROC analysis in the training set and validation data set.
