# Supplementary figures and images for: Identification of immune cell infiltration and diagnostic biomarkers in unstable atherosclerotic plaques by integrated bioinformatics analysis and machine learning
Source: Front Immunol. 2022 Sep 23;13:956078. doi: 10.3389/fimmu.2022.956078 (PMC9537477; doi:10.3389/fimmu.2022.956078)

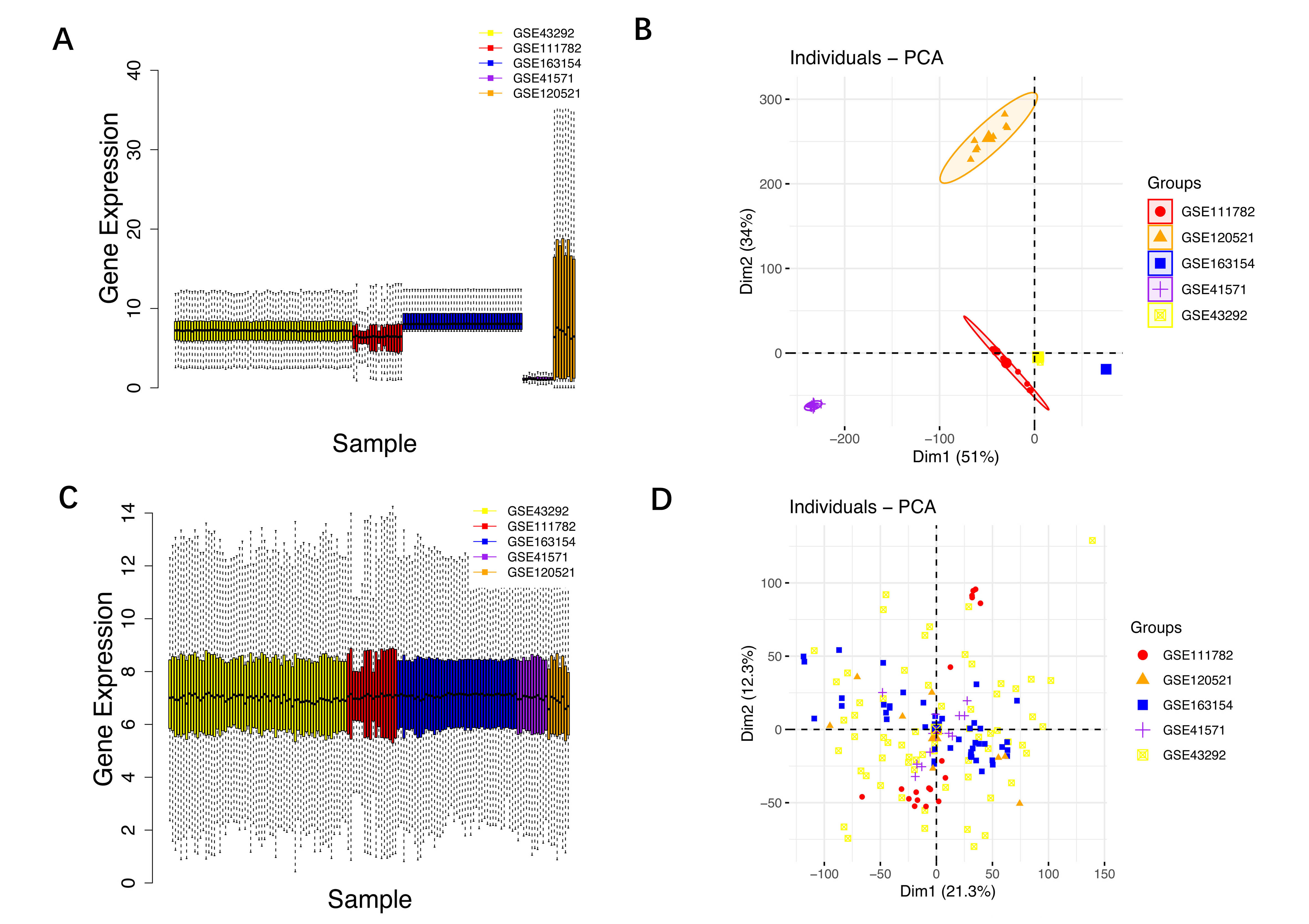

Supplement: Supplementary Figure 1 — Data processing. (A, B) Box plot and principal component analysis (PCA) of expression profile across selected dataset before batch effect correction. (C, D) Box plot and principal component analyses of expression profile after batch effect correction. [file Image_1.jpeg]

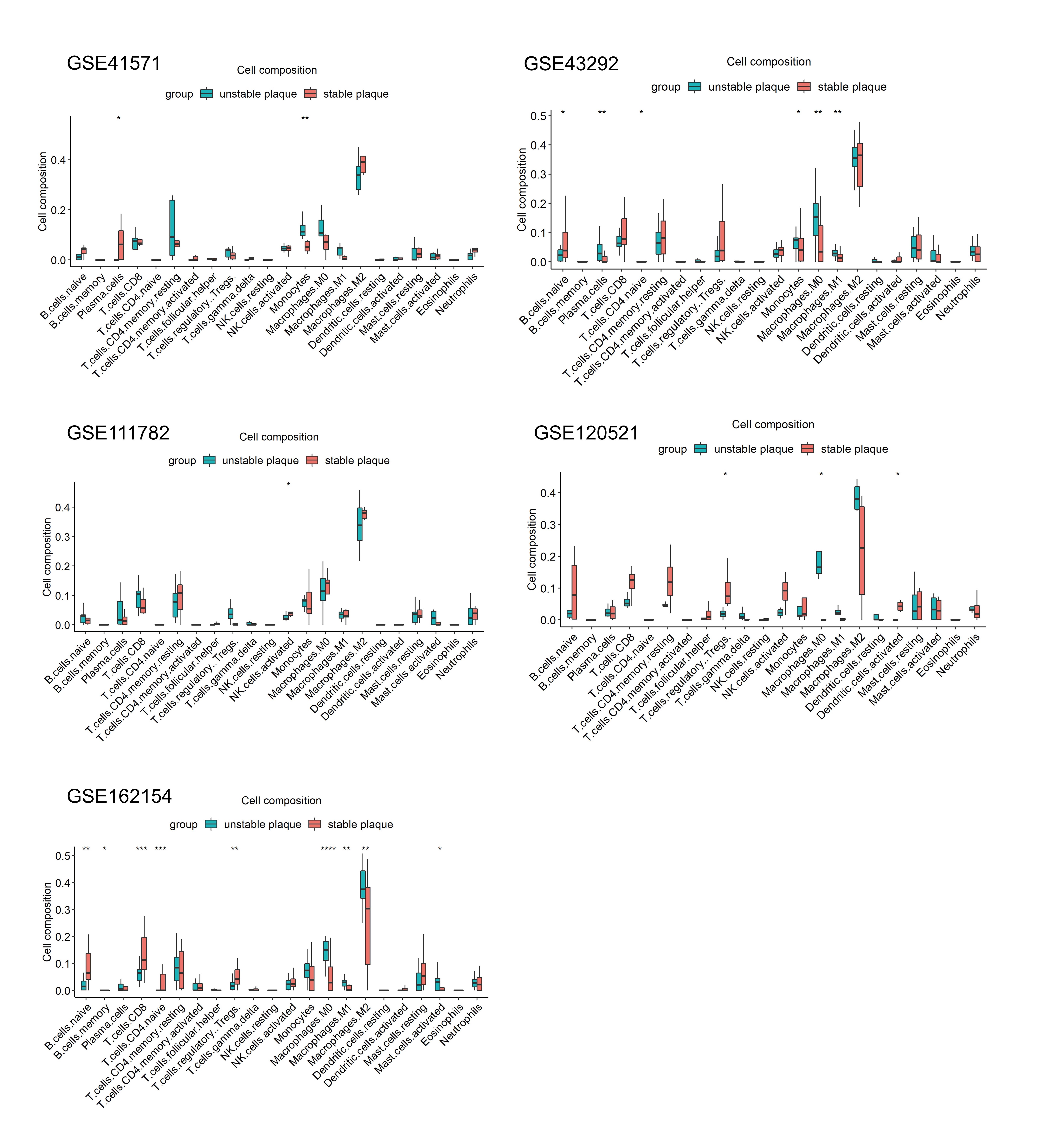

Supplement: Supplementary Figure 2 — Immune infiltration in individual dataset. Identifying the significantly different infiltrates of immune cells in unstable and stable plaques by wilcoxon test [file Image_2.jpeg]

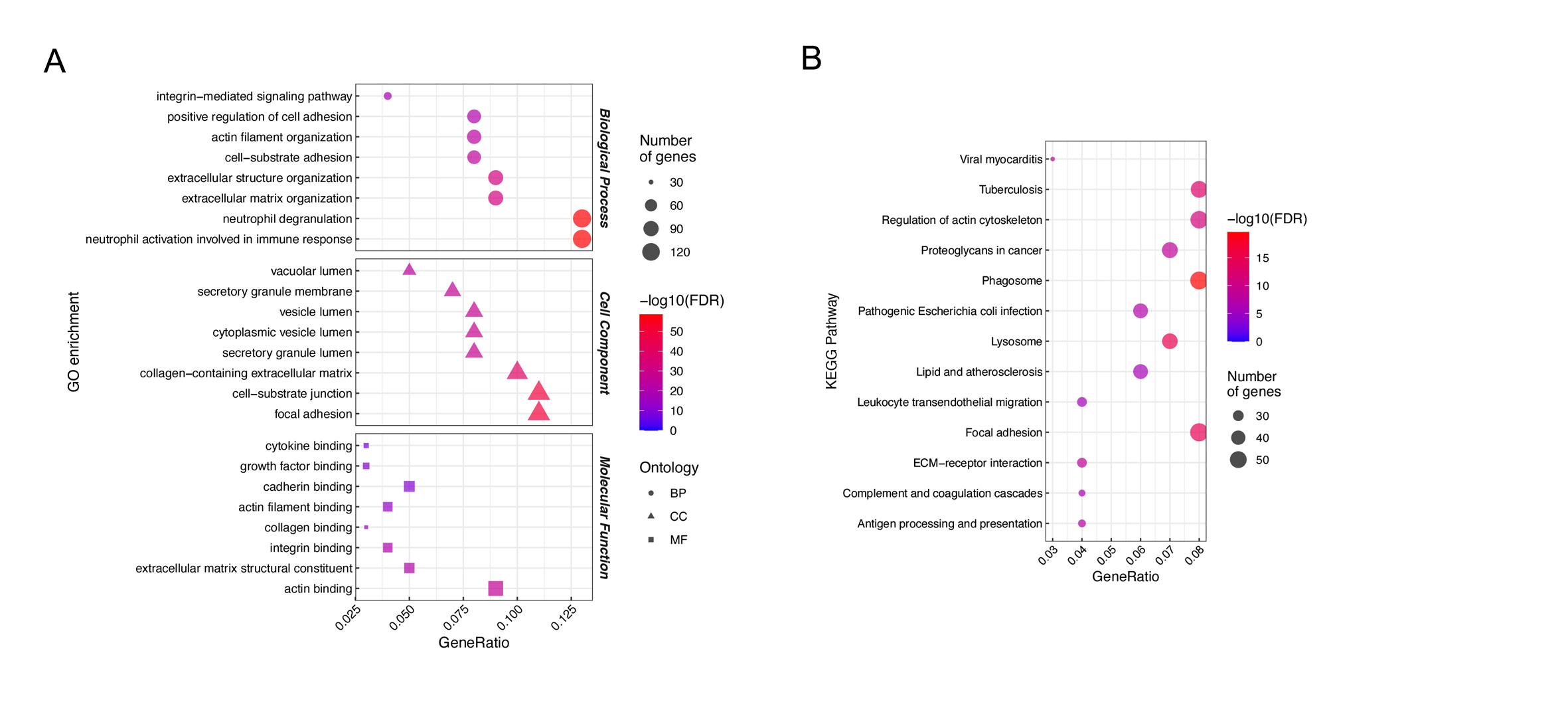

Supplement: Supplementary Figure 3 — GO and KEGG pathway enrichment analysis of the DEGs between unstable and stable plaques. (A) Dot plot of enriched GO termed for DEGs. (B) Dot plot of enriched KEGG pathway for DEGs. [file Image_3.jpeg]

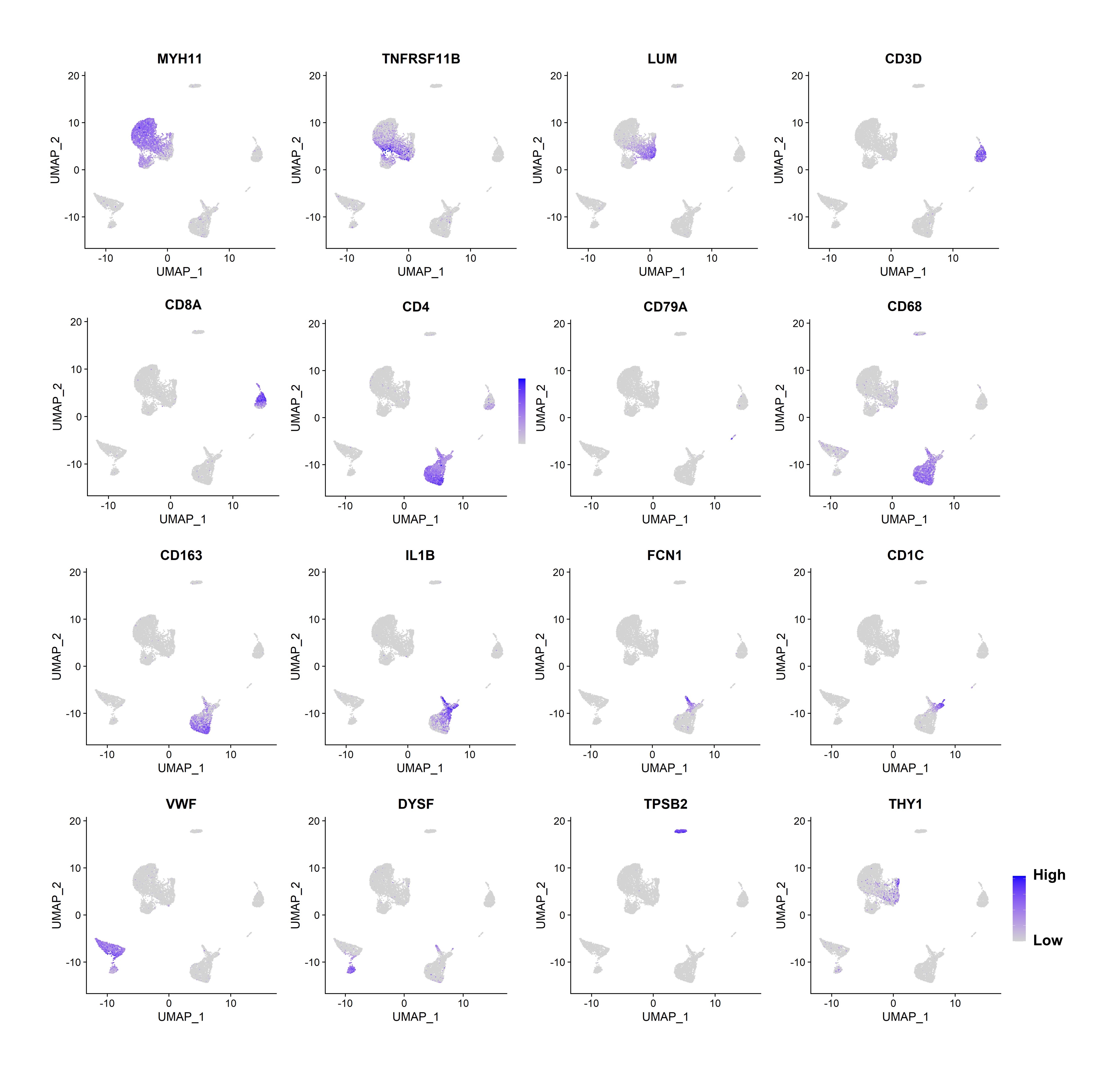

Supplement: Supplementary Figure 4 — Cell Annotation by signature markers in scRNA-seq data. Heatmap of the expression levels of representative marker genes of the atherosclerotic plaque clusters. The color represents the gene expression levels. [file Image_4.jpeg]

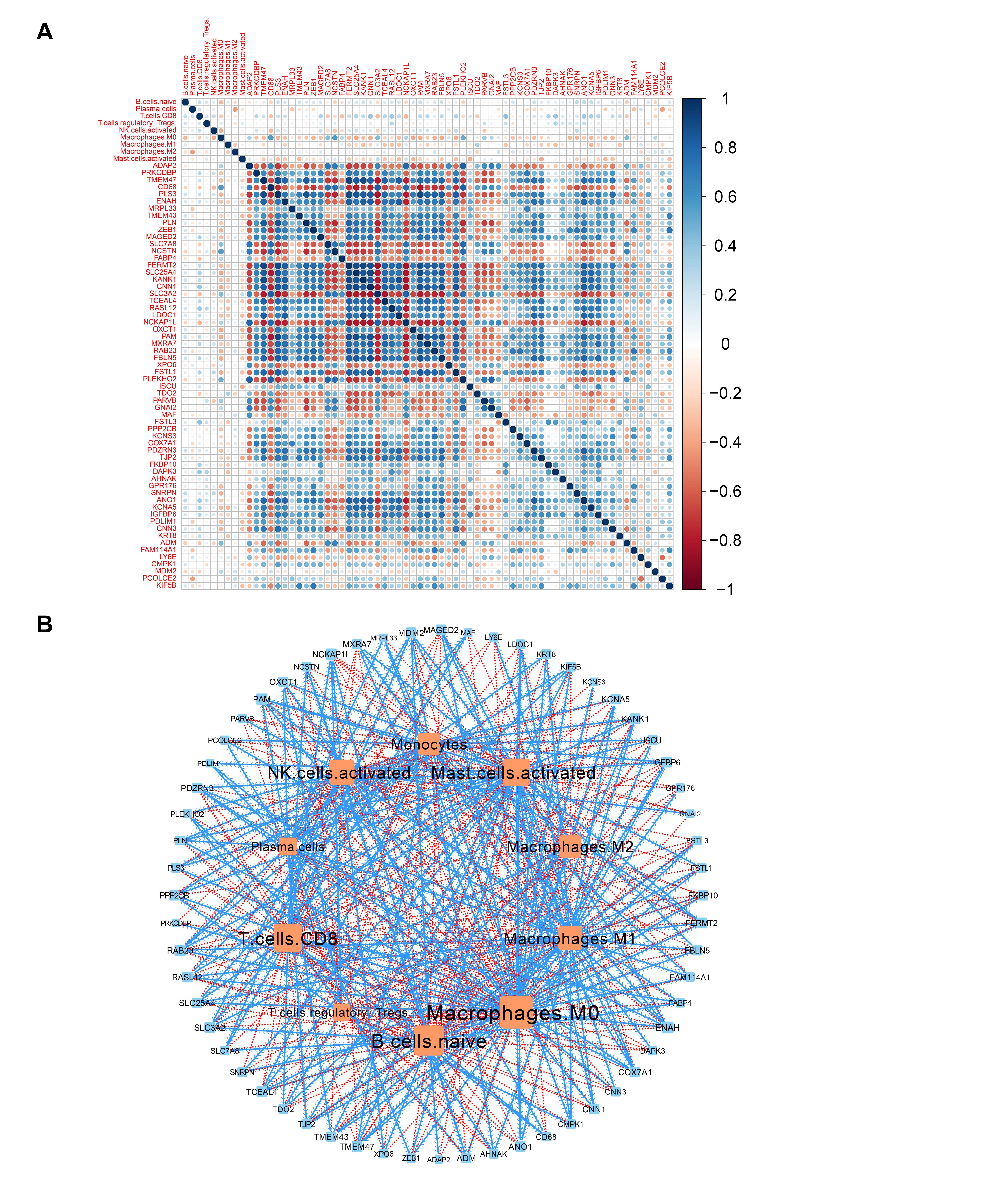

Supplement: Supplementary Figure 5 — Correlation of DEGs and key immune cell types. (A) Heatmap shows Spearman correlation between DEGs and immune cells. (B) Network between key DEGs and key immune cell types. Red lines represent positive correlation while blue ones represent negative correlation. Immune cell types are colored in orange while genes are colored in blue. [file Image_5.jpeg]

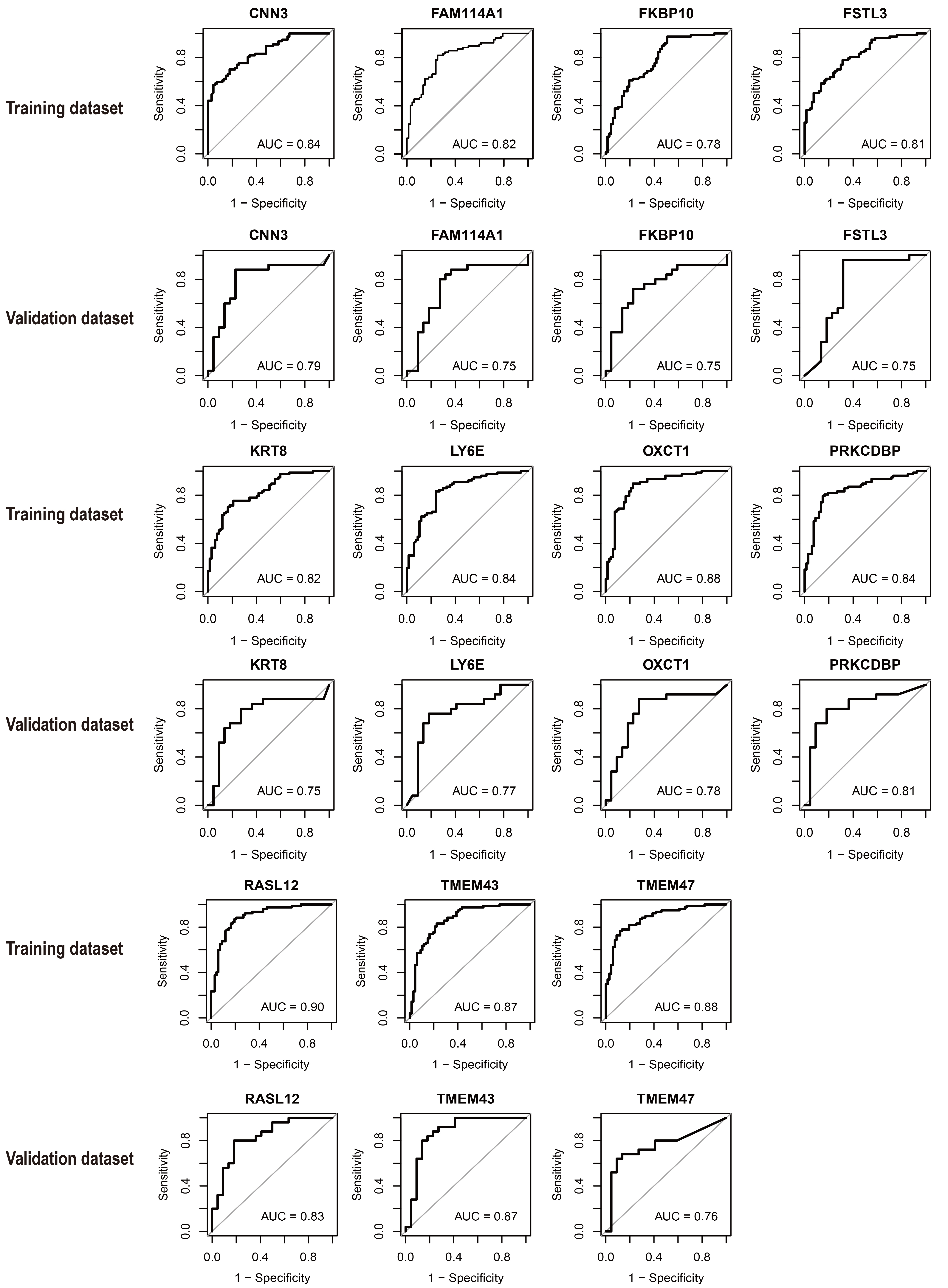

Supplement: Supplementary Figure 6 — Potential biomarkers for unstable plaques. Diagnostic effectiveness of the potential biomarkers by ROC analysis in the training set and validation data set. [file Image_6.jpeg]
